# Expanding genomics of mycorrhizal symbiosis

**DOI:** 10.3389/fmicb.2014.00582

**Published:** 2014-11-04

**Authors:** Alan Kuo, Annegret Kohler, Francis M. Martin, Igor V. Grigoriev

**Affiliations:** ^1^United States Department of Energy Joint Genome InstituteWalnut Creek, CA, USA; ^2^UMR, Lab of Excellence for Advanced Research on the Biology of TRee and Forest Ecosystems, Tree-Microbe Interactions, Institut National de la Recherche Agronomique, Université de LorraineNancy, France

**Keywords:** mycorrhizae, *Laccaria*, *Tuber*, *Rhizophagus*, *Glomus*

## Abstract

The mycorrhizal symbiosis between soil fungi and plant roots is a ubiquitous mutualism that plays key roles in plant nutrition, soil health, and carbon cycling. The symbiosis evolved repeatedly and independently as multiple morphotypes [e.g., arbuscular mycorrhizae (AM), ectomycorrhizal (ECM)] in multiple fungal clades (e.g., phyla Glomeromycota, Ascomycota, Basidiomycota). The accessibility and cultivability of many mycorrhizal partners make them ideal models for symbiosis studies. Alongside molecular, physiological, and ecological investigations, sequencing led to the first three mycorrhizal fungal genomes, representing two morphotypes and three phyla. The genome of the ECM basidiomycete *Laccaria bicolor* showed that the mycorrhizal lifestyle can evolve through loss of plant cell wall-degrading enzymes (PCWDEs) and expansion of lineage-specific gene families such as short secreted protein (SSP) effectors. The genome of the ECM ascomycete *Tuber melanosporum* showed that the ECM type can evolve without expansion of families as in *Laccaria*, and thus a different set of symbiosis genes. The genome of the AM glomeromycete *Rhizophagus irregularis* showed that despite enormous phylogenetic distance and morphological difference from the other two fungi, symbiosis can involve similar solutions as symbiosis-induced SSPs and loss of PCWDEs. The three genomes provide a solid base for addressing fundamental questions about the nature and role of a vital mutualism.

## INTRODUCTION

The roots of most plants form intimate mutualistic associations with soil fungi known as “mycorrhizae.” The mycorrhizal symbiosis is both ancient (among early land plants 410 ma) and pervasive (>80% of plants participate; Tedersoo et al., 2010), and thus underpins most terrestrial ecosystems, the soil portion of the global carbon budget, and much agricultural production (Read and Perez-Moreno, 2003). Mycorrhizae can provide stress tolerance and metal detoxification to the host plant (Hall, 2002), but the fundamental transactional logic of the symbiosis is the exchange of sugar photosynthesized by the plant for phosphorus and other nutrients acquired by the fungus (Martin and Nehls, 2009). A major goal of mycorrhizal studies is to define the symbiosis in molecular terms, i.e., to identify the “symbiosis genes” that encode the molecules that mediate and regulate symbiosis development and interspecific metabolic pathways.

This seemingly straightforward metabolic exchange has evolved many times among many different species pairings and been implemented in a diverse array of structural forms, some extracellular to the root cell and others intracellular but extracytoplasmic. The latter includes arbuscular mycorrhizae (AM), where the fungal hypha penetrates the root cell wall and invaginates (but does not penetrate) the root cell membrane, producing a tree-shaped arbuscule and a large surface area for nutrient exchange. AM is at once more intimate, more dependent (obligate for the fungus), more widespread (most plants can partner), and more ancient (∼410 ma) than other mycorrhizal types. In morphological contrast, ectomycorrhizal (ECM) fungi remain outside of the root cell wall, forming an intercellular hyphal network and a sheath of aggregated hyphae that encases the whole root tip and thus mediates the root’s external interactions with the soil. ECM is the second most common mycorrhizal type, mostly with woody plants. The ECM fungus orders Agaricales, Boletales, and Russulales are at least the same age as the Pinaceae (∼160 ma), suggesting that ECM plausibly evolved at this point. There are other less common or more obscure mycorrhizal types, including orchid mycorrhizae (OM) and ericoid mycorrhizae (ERM), restricted to the Orchidaceae and the Ericaceae (acid-tolerant heathers such as cranberry), respectively. Both OM and ERM have both extra- and intracellular (but not AM) morphological components, but the fungal partner is sometimes capable of switching between morphotypes in a host-dependent manner (Dearnaley et al., 2012).

The morphological and ecological diversity of mycorrhizal fungi is matched by their phylogenetic diversity, encompassing many mushrooms and other fruiting bodies famous for their gastronomy (e.g., porcini, matsutake, chanterelle, morel, truﬄe) or toxicity (e.g., fly agaric). Three of the top-level fungal phyla (Glomeromycota, Ascomycota, and Basidiomycota) have mycorrhizal representatives, but within Basidiomycota and Ascomycota the symbiosis has evolved independently many times in many subclades (66x; Tedersoo et al., 2010). A taxonomic level as low as genus may harbor both symbiotic and non-symbiotic species. Most of the Ascomycota and Basidiomycota symbioses are ECM, the exceptions being ERM Ascomycota and OM Basidiomycota. In contrast, all known Glomeromycota are AM and all known AM are Glomeromycota, suggesting monophyly of both the clade and the symbiosis. The divergence between Glomeromycota and Dikarya (Ascomycota+Basidiomycota) is deep (>800 ma). Glomeromycota have no known sexual cycle. The hyphae and spores are aseptate and multinucleate, with contradictory evidence indicating that some species may or may not be heterokaryotic. Where the Dikarya life cycle is known, Basidiomycota colonize ECM as dikaryons while Ascomycota colonize ECM as monokaryons.

The diversity of mycorrhizae provides motive and opportunity for application of an equally diverse array of investigative methods. Numerous models for physiological, ecological, and molecular biological study have been developed. For example, *in vitro* hyphal-branching assays have been used to isolate plant-secreted small molecules that stimulate AM and ECM fungal morphogenesis (Lagrange et al., 2001; Akiyama et al., 2005) and conversely root-branching and other assays have been used to identify fungus-secreted molecules that promote ECM and AM formation (Nehls et al., 1998; Felten et al., 2009; Splivallo et al., 2009; Maillet et al., 2011). As a second example, stable heterologous gene expression has been accomplished in ECM Basidiomycota (Marmeisse et al., 1992; Hanif et al., 2002; Kemppainen et al., 2005; Pardo et al., 2005), both heterologous expression and gene knockout in ERM Ascomycota (Martino et al., 2007; Abbà et al., 2009), and transient heterologous expression in ECM Ascomycota and AM Glomeromycota (Grimaldi et al., 2005; Helber and Requena, 2008). As a third example, various high-throughput RNA-interrogation methods have been used to recover symbiosis-specific transcripts from *in vitro* models of ECM and AM (Kim et al., 1998; Voiblet et al., 2001; Tamasloukht et al., 2003; Johansson et al., 2004; Duplessis et al., 2005), including a comprehensive multi-sample multi-method analysis of an AM fungal transcriptome that revealed many symbiosis-specific genes, and even meiosis genes in this putatively asexual organism (Lanfranco and Young, 2012; Tisserant et al., 2012).

**FIGURE 1:**
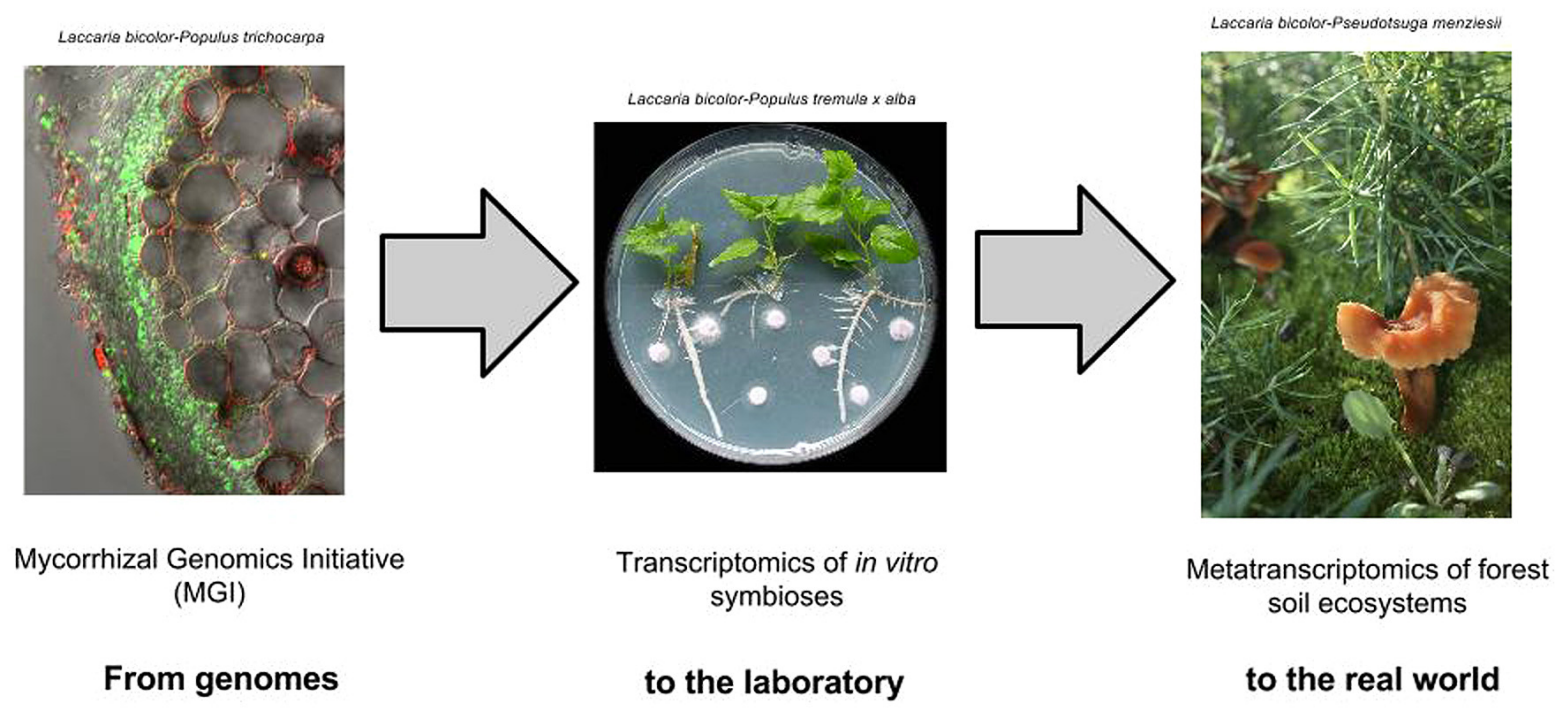
**From sequence to function of symbiotic genes.** Understanding evolution and function of mycorrhizal interactions can be driven by large scale genomics, transcriptomics and metatranscriptomics studies.

The biochemical, genetic, and transcriptomic experiments are being aided by a massive effort to sequence the genomes of multiple mycorrhizal fungal (**Figure 1**). The first three of those genomes to be published are those of the ECM basidiomycete *Laccaria bicolor*, the ECM ascomycete *Tuber melanosporum*, and the AM glomeromycete *Rhizophagus irregularis* (formerly *Glomus intraradices*; Martin et al., 2008, 2010; Tisserant et al., 2013). These first three were chosen for both their diversity and their individual scientific and economic significance. They represent the two most important mycorrhizal morphotypes and three major fungal phyla. The ECM basidiomycete *L. bicolor* and the AM glomeromycete *R. irregularis* were also selected as part of a larger effort to sequence the microbiome of the bioenergy-domesticated poplar tree *Populus trichocarpa*. The ECM ascomycete *T. melanosporum* is of commercial importance in its own right as the gustatory delicacy, black truﬄe.

Each of the three genomes posed significant technical challenges due to their unprecedentedly (at the time of each sequencing project) large size and repetitive nature. All three genomes harbor large numbers of transposable elements (TEs); in addition, *L. bicolor* and *R. irregularis* have very large numbers of gene families, many of which have very large numbers of genes (**Table 1**).

**Table 1:** Properties of the first sequenced mycorrhizal fungal genomes.

Species	*Laccaria bicolor*	*Tuber melanosporum*	*Rhizophagus irregularis*
Phylogeny	Basidiomycota, Agaricales	Ascomycota, Pezizales	Glomeromycota, Glomerales
Mycorrhizal morphotype	Ectomycorrhiza	Ectomycorrhiza	Arbuscular mycorrhiza
Plant partners	Broad range of forest trees, hardwoods and conifers, such as poplars and firs	Narrow range of forest trees, hardwoods and conifers, such as hazel tree and oaks	Hundreds of herbaceous plant species, including crops such as wheat and rice
Genomic assembly (Mbp)	60.7	125.0	91.1
Repeat-masked total (Mbp)	15.1 (25%)	65.3 (52%)	14.0 (15%)
# Predicted genes	23132	7496	30282
Average # exons/gene	5.28	3.87	3.46
# Predicted gene families	3523	799	2749
Average # genes/family	5.02	3.79	8.00
Average protein length (aa)	356	439	270
# Predicted signal peptides	3201 (14%)	1224 (16%)	1995 (7%)
# Distinct Pfam domains	2348	2272	2469
Major publication	Martin et al. (2008)	Martin et al. (2010)	Tisserant et al. (2013)

*All numbers are calculated from the genomes’ computationally reconstructed assemblies and annotations.*

### THE ECTOMYCORRHIZAL BASIDIOMYCETE *Laccaria bicolor*

The genome of *L. bicolor* was the first published of a mycorrhizal fungus (Martin et al., 2008) and led directly to identification of many categories of molecules potentially involved in symbiosis: secreted proteases, lipases, carbohydrate-active enzymes (CAZymes), enzymes for all core carbohydrate metabolic pathways and for fatty acid metabolism (Deveau et al., 2008; Reich et al., 2009), transporters of hexoses and of nitrogenous compounds (López et al., 2008; Lucic et al., 2008), aquaporins (Dietz et al., 2011), multicopper oxidases (Courty et al., 2009), antioxidant enzymes (Morel et al., 2008), signal-transduction protein kinases and small GTPases (Rajashekar et al., 2009), hydrophobins (Plett et al., 2012), and mating-type loci (Niculita-Hirzel et al., 2008). In all these studies the genes were subjected to comparative and phylogenetic analysis with homologs in other fungi (see below) and to transcriptomic analysis (see below). In the carbohydrate and lipase pathway studies, direct assay of storage carbohydrates and fatty acids allowed further pathway reconstruction. In the hexose transporter and aquaporin studies, function was confirmed by genetic complementation of *Saccharomyces* mutants.

Molecular manipulation of the organism complements genomics. Thus it is of great interest that RNA silencing methodology has been developed to knockdown genes in *L. bicolor* (Kemppainen et al., 2009). Such experiments have demonstrated that nitrate reductase and nitrate transporter (Kemppainen and Pardo, 2013), and a mycorrhiza-induced small secreted protein (MiSSP; Plett et al., 2011) are involved in symbiosis.

Complementing these “bottom–up” approaches, a sequenced genome allows application of “top–down” surveys of potential symbiosis genes. Comparison with other Agaricales genomes, at the time all saprotrophic, revealed a large genome size attributable to both TEs and large numbers of large gene families (Martin et al., 2008). Many of these appeared to be lineage-specific, without homologs in the saprobic Agaricales nor Pfam nor other domains allowing straightforward inference of function. Most of the core and potentially symbiosis-related gene families described above are not expanded in *L. bicolor*, with the notable exception of certain families of signal transduction enzymes (Rajashekar et al., 2009). The genome lacks invertase as well as many plant cell wall-degrading enzyme (PCWDE) families, both consistent with notions that *L. bicolor* is dependent on its plant host for carbohydrate and does not activate its host’s defenses (Deveau et al., 2008; Martin and Selosse, 2008; Martin et al., 2008). *L. bicolor* was also the sole mycorrhizal representative in a phylogenomic study of an unprecedentedly large collection of whole genomes to elucidate the evolutionary history of wood decomposition (Floudas et al., 2012). The results suggested that the Agaricomycetes ancestor of *L. bicolor* was a ligninolytic fungus, consistent with the loss of most CAZyme categories by *L. bicolor* as well as non-genome based studies suggesting the repeated, unrelated, and presumptively irreversible adoption of the mycorrhizal lifestyle within many clades of Agaricomycetes (Plett and Martin, 2011; Ryberg and Matheny, 2012; Wolfe et al., 2012).

Transcriptomics enhances genome analysis by showing expression and regulation. Computational prediction of orphan genes and their cleaved signal peptides defined a large set of small secreted proteins (SSPs) in *L. bicolor*. Transcriptomics showed that some of the SSPs are differentially expressed between free-living mycelia and mycorrhizae (Martin et al., 2008). These mycorrhiza-induced SSPs (MiSSPs) appear specific to *L. bicolor*, in that homologs have not been found in other fungi. The 7-kD MiSSP7 is the most highly induced of these genes (>10 k-fold), and has been intensely scrutinized using traditional bottom–up techniques, including immunolocalization, conditional expression studies (Plett and Martin, 2012), and gene knockdown (Plett et al., 2011), all further implicating MiSSP7 as an effector in symbiosis. MiSSP7 interacts with the poplar protein PtJAZ6, a negative regulator of jasmonic acid (JA)-induced gene regulation in poplar (Plett et al., 2014). MiSSP7 protects PtJAZ6 from JA-induced degradation. Furthermore, MiSSP7 blocks or mitigates the impact of JA on *L. bicolor* colonization of host roots. In addition to helping define novel genes, transcriptome data was used to confirm or discover regulation of many of the core and symbiosis-related genes described above, and were also used to validate and correct the genome annotation as a whole (Larsen et al., 2010). In combination with the poplar genome, whole-mycorrhizal transcriptomics was used to attempt to reconstruct a comprehensive metabolic pathway description for the symbiosis (Larsen et al., 2011).

Similarly, proteomics supported the systematic evaluation of the secretome of the saprotrophic phase of *L. bicolor*. Cell-free media from mycelial culture was separated using combinations of isoelectric focusing, gel electrophoresis, and liquid chromatography, and the separated peptides were measured by mass spectrometry and mapped back against the genomic annotation (Vincent et al., 2012). Cleaved signal peptides were predicted in 103 of the secreted proteins, including a limited set of CAZymes, proteases, SSPs, and one MiSSP with a glycophosphatidylinositol anchor. This study was confined to the free-living phase, as the mycorrhizal is so preponderantly plant tissue that current techniques have difficulty detecting the relatively rare fungal proteins.

Other than genes, the genome led directly to a collection of repetitive sequences, including microsatellites (Labbé et al., 2011) and TEs (Labbé et al., 2012). In principle these could be used as markers for population and ecosystem surveys of *L. bicolor in situ*. Already it appears that some microsatellite loci are unstable enough to vary between generations (Labbé et al., 2011). This was also demonstrated with hydrophobin genes located near TEs, suggesting a mechanism for evolutionary change (Plett et al., 2012).

### THE ECTOMYCORRHIZAL ASCOMYCETE *Tuber melanosporum*

The second mycorrhizal fungal genome published was that of *T. melanosporum* (Martin et al., 2010), an ascomycete not related to *L. bicolor* and yet also an ECM. As with *L. bicolor*, the sequenced genome facilitated the identification and analysis of many gene families of interest to symbiosis studies, including CAZymes (induced during mycorrhiza formation, apparently to force a path between the root cells for the mycelium), lipases, multicopper oxidases, an invertase (unlike *L. bicolor*), other carbohydrate metabolism enzymes (Ceccaroli et al., 2011), metal detoxification genes (Bolchi et al., 2011), and cell wall metabolism enzymes (Balestrini et al., 2012; Sillo et al., 2013). As this genome was also the first sequenced for the Pezizomycetes, it also prompted investigation in another fungal clade of genes of general mycological interest, such as cytoskeleton components that determine hyphal morphology (Amicucci et al., 2011). Conversely, the genome also facilitated investigation of traits specific to the life history of *T. melanosporum*, such as sulfur metabolism genes that produce fruiting body (the truﬄe *per se*) volatiles (Martin et al., 2010), cold-shock proteins that may mediate the seasonality of fruiting body development (Zampieri et al., 2011), and tyrosinases and laccases that catalyze melanins implicated in development (Zarivi et al., 2011, 2013). In almost all of the above studies, the genes were subject to both phylogenetic and transcriptomic analyses, some were complemented with microscopic observations (cell wall, cytoskeleton, melanins), and some were confirmed with metabolite or enzyme assays (carbohydrates, metals, melanins).

As with *L. bicolor*, the *T. melanosporum* genome and transcriptome combined are a powerful top–down analytical resource. The transcriptome was used to improve the genomic annotation and to identify alternative and antisense transcripts, and alternative splice variants, some of which are developmentally or symbiotically specific (Tisserant et al., 2011). Conversely, predicted transcription factors (TFs) combined with transcriptomics and a yeast “transcriptional activator trap” system (a variant yeast-2-hybrid screen) identified 29 developmentally regulated TFs (Montanini et al., 2011). Physical separation of mycorrhizal tissues from soil hyphae by microdissection allowed still greater resolution of symbiosis-specific transcripts (Hacquard et al., 2013). As with *L. bicolor*, the *T. melanosporum* genome enabled proteomics by electrophoresis, chromatography, and mass spectrometry (Islam et al., 2013).

The *T. melanosporum* life cycle has not been reconstituted in the laboratory, and only monokaryons have been documented in the wild. The genome revealed putative meiosis genes, as well as a mating locus (Martin et al., 2010), which was used to discover an alternate idiopathic mating locus in the wild (Rubini et al., 2011a). Strains with different mating loci were spatially separated in a single orchard (Rubini et al., 2011b), but this could not be explained by heterokaryon incompatibility, despite the presence of multiple potential HET genes in the genome (Iotti et al., 2012). The genes were not polymorphic among 18 strains. In contrast, the TE- and microsatellite-rich genome has proven to be a rich source of markers for demonstrating the diversity and distribution of populations in the field (Murat et al., 2011, 2013).

### THE ARBUSCULAR MYCORRHIZAL GLOMEROMYCETE *Rhizophagus irregularis*

The third published mycorrhizal genome was that of *R. irregularis* (Tisserant et al., 2013), an AM fungus differing profoundly from the two ECM fungi in morphology and development. Being the first sequenced Glomeromycota, *R. irregularis* is not Dikarya and is even more phylogenetically remote from the other two fungi, with the largest genome encoding the largest gene set. The genome allowed cloning and characterization of a monosaccharide transporter both specific to and required for the symbiosis (Helber et al., 2011). Some signal transduction pathway genes, especially tyrosine kinase-like genes, were expanded (Tisserant et al., 2013). The CAZyme repertoire was more reduced than even that of *L. bicolor*, and there was neither invertase nor sucrose transporter, suggesting even greater dependence of the fungus on its host for carbohydrate. Secondary metabolite gene clusters (polyketide synthases, non-ribosomal peptide synthetases, terpene cyclases, and dimethylallyl tryptophan synthetases) were absent, and the set of predicted secreted effectors also appeared small (Lin et al., 2014). The latter includes five proteins with a putative Crinkler domain characteristic of the Heterokont Oomycota and *Batrachochytrium dendrobatidis* but no other fungi, as well as 13 proteins similar to SP7, a *R. irregularis* effector protein previously cloned using a secretion trap screen (Kloppholz et al., 2011). Transcriptomics revealed a modest set of symbiosis-upregulated genes, including lineage-specific MiSSPs (Tisserant et al., 2013).

Phylogenomics is helping to resolve a long-standing question as to the placement of the Glomeromycota in the fungal tree of life, given their deeply divergent life history and long paleontological record. With the first Glomeromycota genome plus the genomes of many other basal fungi, large numbers of orthologs are available for concatenated multiple sequence alignment and tree building. The most recent efforts point to closer relationship to Mucoromycotina (the classical case of former “zygomycetes”) than to Dikarya, and even closer relationship to Mortierellomycota (Tisserant et al., 2013; Lin et al., 2014).

Cloning of potential meiosis genes and mating type loci in partial genomes (genome survey sequences and transcript sequences of multiple strains) suggest the possibility of a cryptic sexual cycle (Tisserant et al., 2012; Halary et al., 2013; Riley et al., 2014). However, the genome confirms that the genomic contexts of the would-be mating loci are not similar to those of known sexual fungi (Tisserant et al., 2013). In contrast, the genome demonstrates low levels of polymorphism between genomic reads within cells and even between nuclei, thus resolving the long-standing ploidy controversy in favor of homokaryosis.

## CONCLUSION

The first three sequenced genomes of mycorrhizal fungi were groundbreaking. They revealed potential molecular mechanisms underpinning these symbioses, and offered a first glimpse of the evolution of the different types of mycorrhizae. The genome of *L. bicolor* provided the first genetic blueprint of a mycorrhizal fungus, with its expansive genome and proteome. It also enabled other –omics approaches and the identification of the MiSSPs, a novel family of symbiosis effectors. The genome of *T. melanosporum* provided the first comparison between 2 phylogenetically unrelated ECM fungi, contrasting the expanded proteome of *Laccaria* with a compact proteome embedded in the massively enlarged and repetitive genome of *Tuber*. Comparative genomics did not reveal any universal “symbiosis genes,” but did demonstrate convergence of genomic features such as lack of PCWDE and secondary metabolite genes. The genome of *R. irregularis* provided the first opportunity to explore an AM fungus. Despite its great evolutionary distance and morphological distinctiveness from the other two species, the *Rhizophagus* genome showed similar reductions of PCWDEs and expansions of MiSSPs. Thus while the mycorrhizal symbiosis now appears unlikely to be defined by a set of universal “symbiosis genes,” it may be explained by convergent traits that independently and repeatedly evolved. Validation of this conclusion requires a broader sampling to mycorrhizal genomes to be sequenced and followed by transcriptomics studies in established host-mycorrhizal laboratory systems as well as metatranscriptomics of natural environments (**Figure 1**).

## Conflict of Interest Statement

The authors declare that the research was conducted in the absence of any commercial or financial relationships that could be construed as a potential conflict of interest.
